# Supplementary material for: Asynchronous magnetic resonance elastography: Shear wave speed reconstruction using noise correlation of incoherent waves
Source: Magn Reson Med. 2022 Oct 27;89(3):990–1001. doi: 10.1002/mrm.29502 (PMC9792433; doi:10.1002/mrm.29502)
Supplement: Supplementary file 1 — DATA S1 MATLAB code used to generate the numerical simulations of Figure 1 [file MRM-89-990-s002.zip › k-Wave/helpfiles/createCWSignals.html]

createCWSignals :: Functions (k-Wave)


# createCWSignals

Generate array of CW signals from amplitude and phase.

## Syntax

```
cw_signal = createCWSignal(t_array, freq, amp, phase)
cw_signal = createCWSignal(t_array, freq, amp, phase, ramp_length)
```

## Description

`createCWSignals` generates a series of continuous wave (CW) signals based on the 1D or 2D input matrices `amp` and `phase`, where each signal is given by:

```
amp(i, j) .* sin(2 .* pi .* freq .* t_array + phase(i, j));
```

To avoid startup transients, a cosine tapered up-ramp is applied to the beginning of the signal. By default, the length of this ramp is four periods of the wave. The up-ramp can be turned off by setting the `ramp_length` to 0.

Example:

```
% define sampling parameters
f = 5e6;
T = 1/f;
Fs = 100e6;
dt = 1/Fs;
t_array = 0:dt:10*T;

% define amplitude and phase
amp = getWin(9, 'Gaussian');
phase = linspace(0, 2*pi, 9).';

% create signals and plot
cw_signal = createCWSignals(t_array, f, amp, phase);
stackedPlot(cw_signal);
```

## Inputs

|  |  |
| --- | --- |
| `t_array` | 1D vector of time points [s] |
| `freq` | frequency of the CW signal [Hz] |
| `amp` | 1D or 2D matrix of amplitudes [au] |
| `phase` | 1D or 2D matrix of phases [rad] |

## Optional Inputs

|  |  |
| --- | --- |
| `ramp_length` | length of the up-ramp used to reduce start-up transients in periods (default = 4) |

## Outputs

|  |  |
| --- | --- |
| `cw_signal` | matrix of CW signals |

## See Also

`extractAmpPhase`, `toneBurst`
